# Immunosuppressive Activity of Exosomes from Granulocytic Myeloid-Derived Suppressor Cells in a Murine Model of Immune Bone Marrow Failure

**DOI:** 10.3390/ijms241914661

**Published:** 2023-09-28

**Authors:** Ash Lee Manley, Jichun Chen, Wendy Fitzgerald, Xingmin Feng, Neal S. Young

**Affiliations:** 1 Hematology Branch, National Heart, Lung, and Blood Institute, National Institutes of Health, Bethesda, MD 20892, USA; ashlee.manley@nih.gov (A.L.M.); chenji@nhlbi.nih.gov (J.C.); youngns@mail.nih.gov (N.S.Y.); 2 Intracellular Interactions, Eunice Kennedy Shriver National Institute of Child Health and Human Development, National Institutes of Health, Bethesda, MD 20892, USA; fitzgerw@nih.gov

**Keywords:** exosomes, granulocytic myeloid-derived suppressor cells (G-MDSCs), aplastic anemia, bone marrow failure, T-cell proliferation

## Abstract

We previously reported that granulocytic myeloid-derived suppressor cells (G-MDSCs) suppressed T-cell activation and attenuated bone marrow failure (BMF) in a minor histocompatibility (minor-H) antigen mismatched murine aplastic anemia (AA) model. In the current study, we tested the hypothesis that exosomes, a subset of extracellular vesicles, are responsible at least partially for G-MDSCs’ therapeutic efficacy. Indeed, exosomes isolated from GMDSCs (G-MDSC-exos) suppressed CD4^+^ and CD8^+^ T-cell proliferation in vitro and mildly attenuated immune BMF in the minor-H mismatched AA model. G-MDSC-exos treatment significantly increased red blood cells, hemoglobin, and total bone marrow (BM) cells, and moderately reduced BM CD8^+^ T cells. G-MDSC-exos’ effects were associated with upregulations in an array of lymphocyte-suppression-related miRNAs such as hsa-miR-142-5p, miR-19a-3p, and miR-19b-3p in both BM CD4^+^ and CD8^+^ T cells. We concluded that G-MDSC-exos attenuate immune BMF via modulating the delivery of immunosuppressive miRNAs into activated T lymphocytes.

## 1. Introduction

Exosomes are a subset of extracellular vesicles formed via double invagination of the cell plasma membrane through the formation of intracellular multivesicular bodies and are typically 30–200 nm in diameter [1,2,3]. They are active during tissue repair, regeneration, and rejuvenation [4], thus are potentially useful for the treatment of human diseases. In the current study, we explored the immunosuppressive activity of exosomes from granulocytic myeloid-derived suppressor cells (G-MDSC-exos) in vitro in cell culture as well as in vivo in a murine model of immune aplastic anemia (AA) [5]. AA is a type of bone marrow failure (BMF) with characteristic features of marrow hypoplasia and peripheral blood pancytopenia due to the immune destruction of hematopoietic cells [6]. While bone marrow (BM) transplantation and immunosuppressive therapy (IST) with antithymocyte globulin (ATG) and cyclosporine A (CsA) can provide effective treatment for most AA patients, new therapies are still needed, especially for patients who relapse or become refractory to IST [6,7,8].

AA has been modeled in mice through allogeneic lymph node (LN) cell infusion from C57BL6 (B6) donors into minor histocompatibility (minor-H) antigen mismatched C.B10-H2b/LilMcd (C.B10) [5] or with major histocompatibility complex (MHC) mismatched CByB6F1 or B6D2F1 recipients [9]. These models are useful for the investigation of AA/BMF pathophysiology [10] and for the testing of therapeutic interventions [5,11]. We used these models to test granulocytic myeloid-derived suppressor cells (G-MDSCs) as a new cell therapy and found that G-MDSC therapy is effective in the treatment of murine AA in the minor-H mismatched LN-cell-infusion-induced BMF model [12].

Encouraged by recent reports of exosome functionality and treatment effects [4,13,14,15,16], specifically by findings showing G-MDSC-exos suppressed immune responses and attenuated disease in animal models of colitis [17,18,19], and that G-MDSC-exos augmented delivery of miRNA to CD4^+^ T cells in a mouse model of rheumatoid arthritis [17], we reasoned that the therapeutic efficacy of G-MDSCs in murine AA/BMF might be partially mediated by G-MDSC-exos. We isolated exosomes from mouse G-MDSCs and tested G-MDSC-exos’ functionality in a lymphocyte proliferation assay in vitro and in an immune BMF model in vivo. BM CD4^+^ and CD8^+^ T cells were isolated and changes in the corresponding miRNA expression were analyzed by comparing BMF mice with and without G-MDSC-exos treatment. Observations from our study illustrate the G-MDSC-exos’ immunomodulatory activity in suppressing T-cell proliferation in vitro and attenuating immune BMF in vivo by the delivery of immunosuppressive miRNAs into activated T cells.

## 2. Results

### 2.1. G-MDSC-exos Confirmation and Quantification

**Figure 1:**
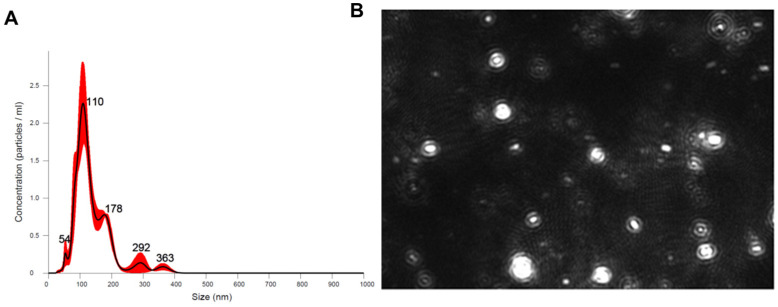
Confirmation of exosomes using a nanoparticle tracking analyzer. Concentration and exosome size are depicted (**A**). The numbers in black represent the size of the exosome, corresponding to peaks in concentration. An image was taken of the exosomes during analysis using a nanoparticle tracking analyzer (**B**).

As illustrated, we used nanoparticle tracking analysis to confirm and quantify isolated G-MDSC-exos isolated from the BM cells of B6 mice and found the mean size of G-MDSC-exos to be 139 nm with a median of 99.8 nm and standard deviation of 61.3 nm (Figure 1A). These data are consistent with the size range of exosomes of 30–200 nm [2]. From the supernatant of 100 × 10^6^ cultured G-MDSC cells, we found 100 *×* 10^8^ exosomes were released, meaning an average of 100 exosomes from each G-MDSC during the 48 h culture. The nanoparticle tracking analysis allowed for the visualization of the G-MDSC-exos (Figure 1B).

### 2.2. Suppression of T-Cell Proliferation In Vitro

**Figure 2:**
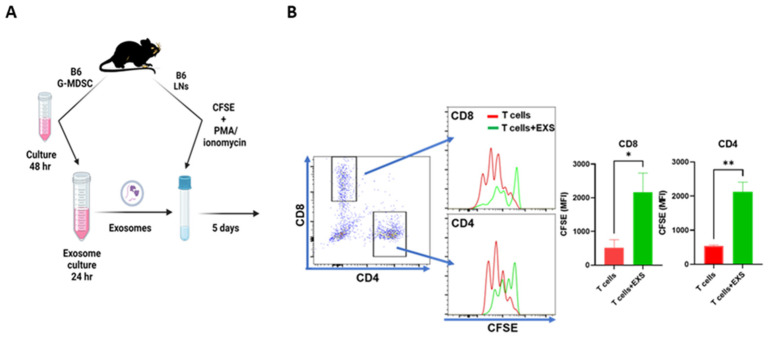
Suppressive effects of G-MDSC exosomes on T-cell proliferation in vitro. (**A**) Preparation of GMDSC exosomes. G-MDSCs were isolated from the bone marrow of B6 mice and then cultured for 48 h at 37 °C with 5% CO_2_ in complete RPMI1640 media for exosome release. The supernatant containing exosomes was cocultured with EXO-TC precipitation solution for 24 h at 4 °C to capture the exosomes. (**B**) Functional assay of exosomes on T-cell proliferation. Isolated exosomes from 20–30 × 10^6^ BM G-MDSCs were cocultured with carboxyfluorescein succinimidyl ester (CFSE)-labeled B6 lymph node (LN) cells (1 × 10^6^/mL each reaction) stimulated with 50 ng/mL phorbol 12-myristate 13-acetate (PMA) and 500 µM ionomycin for 5 days in RPMI 1640 medium supplemented with 10% fetal calf serum and antibiotics. T-cell proliferation is indicated by the decrease in CFSE dye intensity as measured using median fluorescence intensity (MFI). The plots show one of two similar experiments. *, *p* < 0.05; **, *p* < 0.01.

We first tested G-MDSC-exos’ immunosuppressive activity in vitro when LN cells from B6 donor mice were carboxyfluorescein succini-midyl ester (CFSE)-labeled, stimulated using phorbol 12-myristate 13-acetate (PMA) and ionomycin, and cultured for 5 days in complete RPMI 1640 media with or without the addition of G-MDSC-exos (Figure 2A). Relative to the LN cell culture without exosomes (T cells), the addition of G-MDSC-exos (T cells + EXS) significantly increased the retention of CFSE dye in both CD4^+^ and CD8^+^ T cells (Figure 2B). This experiment was performed twice, and similar levels of CFSE-dye retention were detected in response to G-MDSC-exos, indicating that G-MDSC-exos suppressed the proliferation of both CD4^+^ and CD8^+^ T cells upon stimulation with PMA and ionomycin.

### 2.3. Attenuation of Immune BMF In Vivo

**Figure 3:**
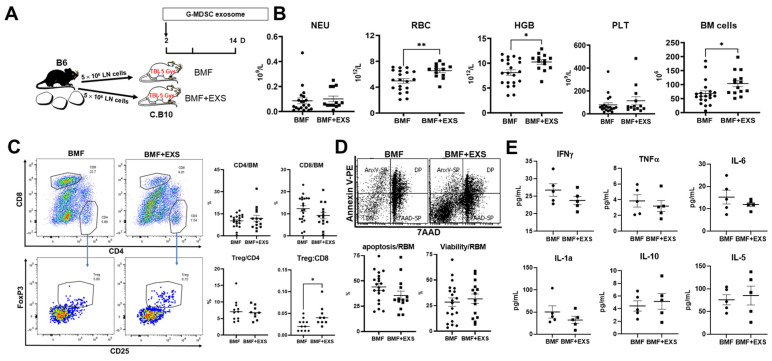
G-MDSC exosomes attenuated immune-mediated bone marrow failure (BMF). (**A**) Male C.B10 mice were preirradiated with 5 Gys total body irradiation and infused with 5 × 10^6^ lymph node (LN) cells/mouse from B6 male donors to induce BMF. Recipient mice were untreated (BMF, N = 20) or were treated with exosomes released from 12–18 × 10^6^ B6 BM G-MDSCs following 48 h of culture in vitro (BMF + EXS, N = 14). (**B**) Mice were bled and euthanized on days 14–17 following LN-cell infusion, and peripheral blood was analyzed for neutrophils (NEUs), red blood cells (RBCs), hemoglobin (HGB), and platelets (PLTs), while the recovery of total BM cells was estimated based on marrow harvested from two tibiae and two femurs. (**C**) Proportions of BM CD4^+^, CD8^+^ T cells, regulatory T cells (Treg) in CD4^+^ T cells, and the ratio of Treg:CD8^+^ T cells were measured via flow cytometry shown as representative dot plots and individual observation. (**D**) Proportions of apoptosis and viable residual bone marrow (RBM, excluding CD4^+^ and CD8^+^ T cells) were measured by flow cytometry shown as representative dot plots and individual observations. Data were combined from three separate experiments. (**E**) Cytokines in available plasma samples were measured by Luminex. *, *p* < 0.05; **, *p* < 0.01.

The G-MDSC-exos’ immunosuppression was further tested in vivo in a B6 => C.B10 LN cell minor-H mismatched AA/BMF model via infusing G-MDSC-exos from B6 donors into C.B10 BMF mice two days after BMF induction (Figure 3A). At days 14–17 after LN cell infusion, the treated recipient mice had significant improvements in red blood cells (32%⇑, *p* < 0.01), hemoglobin (26%⇑, *p* < 0.05), and total BM cells (52%⇑, *p* < 0.05) relative to the controls (Figure 3B). Platelet counts also increased by 45% following G-MDSC-exos therapy, but the change was not statistically significant due to individual variations (Figure 3B). In the BM, the G-MDSC-exos-treated mice had no change in the proportion of CD4^+^ T cells and a trend of decreased CD8^+^ T cells. There was no change in the frequency of regulatory T cells in CD4^+^ T cells, but the ratio of Treg:CD8 was significantly increased after G-MDSC-exos treatment (Figure 3C). The G-MDSC-exos treatment slightly reduced apoptosis (37%⇓, *p* > 0.05) and increased viable cells in residual bone marrow (RBM, excluding CD4 and CD8 T cells; Figure 3D). G-MDSC-exos treatment tended to decrease inflammatory/Th1 cytokines, including IFNγ, TNFα, IL-6, and IL-1a, while increasing Th2 cytokines (IL-10, IL-5), but these changes did not reach statistical differences (Figure 3E). Thus, G-MDSC-exos exerted therapeutic effects in the minor-H mismatched AA/BMF model via attenuating BM-cell apoptosis/destruction and alleviating blood pancytopenia.

### 2.4. Modulation of miRNA Delivery into CD4^+^ and CD8^+^ T Cells

**Figure 4:**
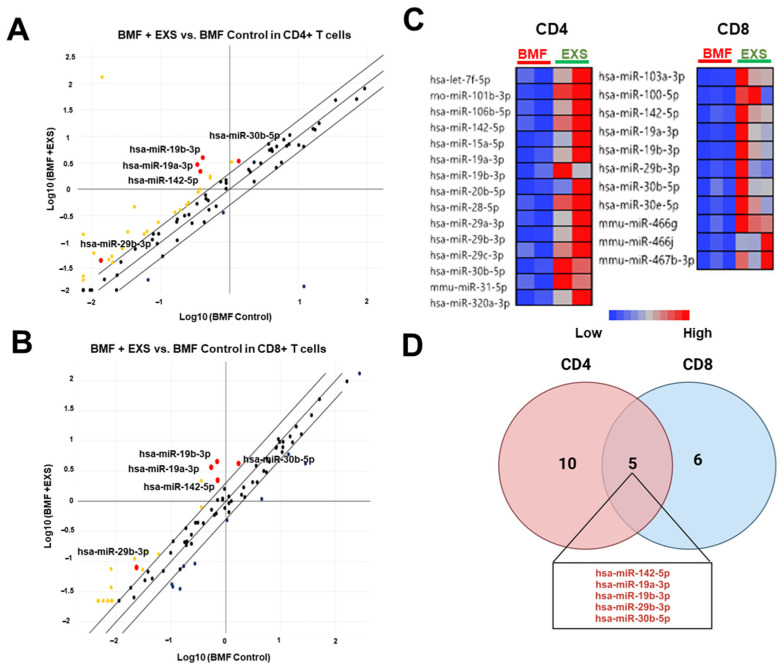
Distinct miRNA profiles CD4^+^ and CD8^+^ T cells from exosome-treated bone marrow failure (BMF) mice. Scatter plots illustrating relative expression levels of 84 lymphocyte-activation-focused miRNAs in BM CD4^+^ (**A**) and CD8^+^ (**B**) T cells using pooled samples from BMF + EXS mice and BMF mice, respectively. The x-axis represents estimated expression differences measured in log10 for BMF control samples, and the y-axis represents estimated expression differences measured in log10 for BMF + EXS. Diagonal lines show a 2-fold expression difference between the two groups. Yellow dots indicate differentially expressed miRNAs in CD4^+^ or CD8^+^ T cells between two groups; red dots differentially expressed miRNAs overlapped between CD4^+^ and CD8^+^ T cells in two groups; black dots are miRNAs not differentially expressed between two groups. (**C**) Heat maps of differentially expressed miRNAs in CD4^+^ (2 pools) and CD8^+^ (3 pools) T cells between two groups. The red–blue color scale indicates normalized miRNA expression levels (red: high expression, blue: low expression). (**D**) The overlap of upregulated miRNAs between CD4^+^ and CD8^+^ T cells is represented using a Venn diagram.

To explore the mechanism of G-MDSC-exos’ efficacy in BMF, we analyzed miRNAs in BM CD4^+^ and CD8^+^ T cells from G-MDSC-exos-treated mice compared with the BMF controls. An array of miRNAs was upregulated in both CD4^+^ and CD8^+^ T cells in G-MDSC-exos-treated mice with changes >2 fold compared with the BMF controls (Figure 4A,B). A total of 15 miRNAs were upregulated in CD4^+^ T cells, and 11 miRNAs were upregulated in CD8^+^ T cells from the BMF+EXS group compared with the BMF control (Figure 4C). CD4^+^ and CD8^+^ T cells both had upregulations in the same five miRNAs: hsa-miR-142-5p, hsa-miR-19a-3p, hsa-miR-19b-3p, hsa-miR-29b-3p, and hsa-miR-30b-5p in BMF + EXS mice compared with controls (Figure 4D).

## 3. Discussion

G-MDSC-exos exerted immunosuppressive activity based on analyses performed in vitro and in vivo. Our observations of G-MDSC-exos’ ability to suppress the proliferation of CD4^+^ and CD8^+^ T cells in vitro and of G-MDSC-exos-treatment-related hematopoietic improvement in the AA/BMF mouse model are consistent with the findings of previous reports showing that G-MDSC-exos treatment reduced the percentages of Th1 and Th17 cells in an arthritis model [17] and suppressed CD4^+^ T-cell proliferation and Th1-cell response in a colitis model, affirming G-MDSC-exos’ immunosuppressive function [18]. More specifically, our current observations are in agreement with those of our earlier report, showing that G-MDSC therapy could mitigate hematopoietic destruction in the same minor-H mismatch immune AA/BMF model [12]. Exosomes derived from G-MDSCs attenuated murine BMF in a similar way to G-MDSCs but were less effective. We speculate that the synergistic action of reactive oxygen species (ROS) and nitrogen species from G-MDSCs and miRNAs from exosomes may contribute to G-MDSCs’ immunosuppression. Exosomes are only part of the components of G-MDSCs and thus only partially contribute to G-MDSC’s effects. Our results are also similar to those in other disease models, confirming the immunosuppressive functional activities of G-MDSC-exos [17,18,19,20].

We previously reported that hsa-miR-142-5p, miR-19a-3p, miR-19b-3p, miR-29b-3p, and miR-30b-5p were downregulated in the CD4^+^ and CD8^+^ T cells of BMF mice relative to control mice [21]. The upregulation of these miRNAs in CD4^+^ and CD8^+^ T cells in BMF mice after treatment in the current study is consistent with the immunosuppressive effects of G-MDSC-exos, thus attenuating BMF. In systemic lupus erythematosus, the inhibition of miR-142-5p led to CD4^+^ T-cell activation [22]. In addition, the downregulation of hsa-miR-29b-3p is associated with IFN-γ production and enhanced inflammation in seasonal allergic rhinitis [23]. Our observation of the upregulation of hsa-miR-29b-3p in G-MDSC-exos-treated mice is in agreement with its suppressive property of inflammation. We could not exclude the possibility that G-MDSC-exos also regulate CD4^+^ and CD8^+^ T cells through indirect effect from other immune cells and/or microenvironment; for example, the cytokines we observed in this study might also be involved in this process.

As a specific type of extracellular vesicle, the small size of exosomes makes them potential carriers for drug delivery [3]. Exosomes carry intracellular components and can exert functional characteristics similar to those of the cells from which they were derived. For example, exosomes derived from myoblasts and mesenchymal stromal cells augmented the repair and regeneration of skeletal and cardiac cells [13,15]. We suggest that G-MDSC-exos exerted an immunosuppressive function, at least partly via modulating the delivery of miRNAs into activated CD4^+^ and CD8^+^ T cells.

## 4. Materials and Methods

### 4.1. Animals and Exosome Preparation

Inbred B6 and congenic C.B10 mice originally obtained from the Jackson Laboratory (Bar Harbor, ME, USA) were bred and housed at a specific-pathogen-free National Institutes of Health animal facility with standard care and nutrition. Mice were used at 2–5 months of age and all animal studies were preapproved by the Animal Care and Use Committee at the National Heart, Lung, and Blood Institute.

G-MDSCs were isolated from B6 BM cells using Ly6G microbeads and magnetic columns (Miltenyi Biotec, Auburn, CA, USA) as previously described [13]. The purity of G-MDSCs was confirmed via flow cytometry to be between 94% and 97% cells based on the CD11b^+^Ly6G^+^Ly6C^low^ phenotype. Collected G-MDSCs were incubated at 37 °C with 5% CO_2_ for 48 h in complete RPMI 1640 medium (RPMI 1640 medium supplemented with 100 U/mL penicillin, 100 U/mL streptomycin, 292 µg/mL L-glutamine, Life Technologies Inc. Carlsbad, CA, USA). Exosomes were prepared from G-MDSC cell culture supernatant using ExoQuick-TC precipitation solution (SBI System Bioscience, Palo Alto, CA, USA) following the manufacturer’s instructions [20]. In brief, ExoQuick-TC precipitation solution was mixed well with G-MDSC culture supernatant at a 1:5 ratio in a sterile vessel and refrigerated overnight at 4 °C. The G-MDSC culture supernatant was centrifuged (1500× *g*, 30 min, 4 °C), resulting in an exosome pellet. We used nanoparticle tracking analysis (NTA, NanoSight NS300 with NTA 3.4 software, Malvern Panalytical, Ltd., Salisbury, UK) to confirm and quantify isolated exosomes. G-MDSC-exos were resuspended in PBS for functional assay in vitro and were resuspended in USP saline for BMF treatment in vivo.

### 4.2. T-Cell Proliferation In Vitro

T-cell proliferation was measured using CFSE dye dilution as reported previously [11]. In brief, LN cells from B6 donors were prelabeled with CFSE dye (Invitrogen, Waltham, MA, USA), stimulated with PMA (Sigma-Aldrich, St. Louis, MO, USA) and ionomycin (Invitrogen) at concentrations of 50 ng/mL and 500 µM, respectively, and cultured for 5 days at 37 °C with 5% CO_2_ in complete RPMI 1640 medium with or without G-MDSC-exos. CFSE-labeled LN cells were stained for CD4^+^ and CD8^+^ T cells and analyzed via flow cytometry to evaluate CFSE dye dilution.

### 4.3. Induction of AA/BMF and G-MDSC Exosome Therapy

BMF was induced as described [5,9,10,11,12,13]. In brief, LN cells from inguinal, axillary, and lateral axillary LNs of B6 donors were washed and injected into sex-matched minor-H mismatched C.B10 recipients through lateral tail vein at 5 × 10^6^ cells/recipient to induce BMF. All recipients received 5 Gys total body irradiation (from a Gammacell 40 source, MDS Nordion, Ottawa, ON, Canada) 4–6 h earlier. G-MDSC-exos were injected into some AA/BMF mice two days later, with each recipient receiving exosomes from 12–18 × 10^6^ cultured G-MDSCs.

### 4.4. Cell Counts and Flow Cytometry

At days 14–17 after BMF induction, blood was collected from the retro-orbital sinus for complete blood counts using an Element HT5 hematology analyzer (Heska Corporation, Loveland, CO, USA). Recipients were euthanized, and BM cells were flushed from bilateral tibiae and femurs, filtered through 85 µm nylon mesh, and counted with a Vi-Cell counter (Beckman Coulter, Miami, FL, USA). Blood and BM cells were stained using an antibody cocktail for flow cytometry analysis. Monoclonal antibodies for mouse CD4 (clone GK 1.5), CD8 (clone 53-6.72), CD11b (clone M1/70), Ly6C (clone HK1.4), Ly6G (clone 1A8), CD25 (clone 3C7), and FoxP3 (clone 150D) were obtained from Biolegend (San Diego, CA, USA). We purchased 7AAD and Annexin V from BD Biosciences (San Diego, CA, USA). Stained cells were acquired using BD FACSCanto II and BD LSRFortessa flow cytometers operated using FACSDiva software (https://www.bdbiosciences.com/en-us/products/software/instrument-software/bd-facsdiva-software. Becton Dickson, San Diego, CA, USA), and flow cytometry data were analyzed using Flowjo software v10.9.0 (Ashland, OR, USA).

### 4.5. Cytokine Measurement

Cytokines in plasma samples were measured using a Luminex mouse magnetic bead panel (R&D systems, Minneapolis, MN, USA) according to the manufacturer’s instructions.

### 4.6. Cell Sorting and RNA Isolation

BM CD4^+^ and CD8^+^ T cells were sorted in the Flow Cytometry Core at the National Heart, Lung, and Blood Institute (NHLBI, Bethesda, MD, USA). Total RNAs, including miRNAs and other small RNAs, were isolated from sorted CD4^+^ and CD8^+^ T cells using miRNeasy Tissue/Cells Advanced Kits (QIAGEN, Germantown, MD, USA). RNA concentration was measured using a Nanodrop device (Peqlab, Erlangen, Germany).

### 4.7. Quantitative Real-Time RT-PCR (RT-qPCR)

RT-qPCR was conducted to quantify miRNA expression. Reverse transcription was performed on an equal amount of RNA from BM CD4^+^ and CD8^+^ T-cell samples using a miRCURY LNA RT kit (QIAGEN), followed by a T-cell and B-cell activation pathway-focused miRNA PCR array per the manufacturer’s instructions. miRNA data were analyzed using QIAGEN analysis software (https://dataanalysis2.qiagen.com/miRCury), and Ct values were normalized using reference miRNAs. miRNA values were considered different if the individual normalized Ct values didn’t overlap and had a fold change of greater than 2 between BMF and BMF+G-MDSC-exos treatment groups. A heatmap was generated using JMP (SAS Institute, Cary, NC, USA) based on normalized expression levels of individual miRNAs.

### 4.8. Statistics

Data were analyzed using standard variance and multiple comparisons using GraphPad Prism statistical software v9 (Boston, MA, USA). Results are presented as means with standard errors. Statistical significance was defined as *p* < 0.05.

## 5. Conclusions

Our study provides fresh evidence showing that G-MDSC-exos behave similarly to G-MDSCs and are capable of alleviating immune BMF in a minor-H mismatched setting through delivery of miRNAs into CD4^+^ and CD8^+^ T cells. We conclude that G-MDSC-exos could be a potential therapy for AA/BMF.

## Data Availability

Data sharing is not applicable to this article.
